# Colorectal cancer in the 45-to-50 age group in the United States: a National Cancer Database (NCDB) analysis

**DOI:** 10.1007/s00464-021-08929-6

**Published:** 2021-12-09

**Authors:** Thais Reif de Paula, Eric M. Haas, Deborah S. Keller

**Affiliations:** 1 Department of Biomedical Sciences, University of Houston College of Medicine, Houston, TX USA; 2 Houston Colon PLLC, Houston, TX USA; 3 Division of Colon and Rectal Surgery, Houston Methodist Hospital, Houston, TX USA; 4 Division of Colorectal Surgery, Department of Surgery, University of California, Davis Medical Center, 2335 Stockton Blvd. NAOB 6322, Sacramento, CA 95817 USA

**Keywords:** Colorectal cancer (CRC), Early-onset colorectal cancer (EOCRC), Screening guidelines, Colonoscopy, Disability, Productivity

## Abstract

**Background:**

Amid increasing awareness of early-onset colorectal cancer (CRC), guidelines in the United States (US) recently lowered the recommended routine CRC screening age from 50 to 45 in average-risk individuals. There are little data on the number of patients in this age group diagnosed with CRC prior to these changes. Our objective was to audit the historic CRC case trends and impact of CRC in the 45-to-50-year-old category prior to new screening recommendations.

**Methods:**

Colorectal adenocarcinoma cases in 45-to-50-year-old patients were queried from the NCDB (2004–2017). Cases were stratified by sex, race, and site. The disability-adjusted lost years (DALY) and lost earnings were estimated. The average annual percentage changes (AAPC) of CRC incidence were estimated using jointpoint analysis. The main outcome measures were DALY and lost earnings. Secondary outcome measures were the 2004–2017 AAPC and the cumulative incidence of potential CRC cases in the 45-to-50 cohort through 2030 without guideline changes.

**Results:**

67,442 CRC patients in the 45-to-50 demographic were identified. The CRC burden resulted 899,905 DALY and $17 billion in lost earnings. The 2004–2017 AAPC was 1.6%, with an estimated 13-year increase of 25%. There were sex-, race-, and anatomic site-specific discrepancies with estimated 13-year increases of 30% for males, 110% for American Indian/ Alaska Natives/ Asian American/ Pacific Islander races, and 31% for rectal cancer by 2030.

**Conclusion:**

CRC has been steadily increasing in the 45-to-50 age group, with tremendous disability and cost ensuing. There is great potential benefit from lowering the recommended routine CRC screening age to 45. Targeted intervention could ensure the most vulnerable segments benefit from the new guidelines, in both reducing the incidence and improving survivorship in CRC patients.

**Graphical abstract:**

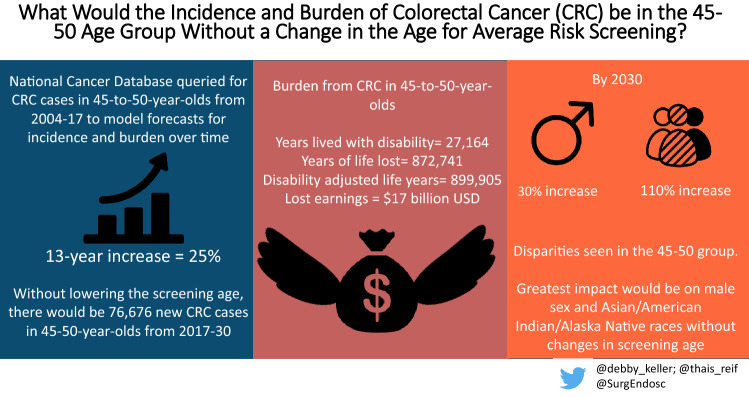

**Supplementary Information:**

The online version contains supplementary material available at 10.1007/s00464-021-08929-6.

The global burden of cancer continues to steadily rise and now is the second leading cause of death, years of life lost (YLL), and disability-adjusted life years (DALY) worldwide [1]. Colorectal cancer (CRC) specifically accounts for a large percent of the global burden, serving as the fourth most incident and second most deadly cancer. There are disparities in the annual CRC trends regionally with age-, socio-economic, and geographic-specific variations. In high-income countries, like the United States (US), increased awareness and resources for screening CRC has reduced the overall incidence. However, the CRC incidence in patients 20-to 49-year-old (early-onset CRC, EOCRC) continues to exponentially rise [2–6]. By estimations, CRC will lead cancer-related deaths in US by 2040 for adults aged 20-to-49 [7].

With these trends, the US Preventive Services Task Force (USPSTF) and the American Cancer Society (ACS) lowered the recommended CRC screening age from 50 to 45 in average-risk individuals in 2020 [8, 9] The effectiveness of this important public health measure depends on awareness and adherence to the guideline. Currently, overall compliance with recommended CRC screening is poor, estimated at 66% nationally [10]. While guidelines provide availability to screening for EOCRC, awareness of the problem and its potential impact may be needed for compliance with recommended screening. To date, there are little data on the incidence and impact of CRC in patients 45-to-50 prior to the new initial screening age recommendation, nor on trends for sex, race, and anatomic location in this population that could be used for targeting screening interventions.

The goal of this work was to audit CRC trends in the 45-to-50-year-old population and the burden of CRC in this population prior to screening recommendation changes. Secondarily, future trends in CRC incidence and disability in the 45-to-50 population without the screening guideline amendment were forecasted through 2030. The hypothesis was that the trends could establish a benchmark for the greatest-risk subgroups to target screening.

## Methods

### Study population

The National Cancer Database (NCDB) was reviewed for colorectal adenocarcinoma cases (C18-20, C26) diagnosed between 2004 and 2017. Included criteria were patients ≥ 45 to < 50 years old at diagnosis and histopathologic confirmation of adenocarcinoma, by International Classification of Diseases for Oncology, 3rd Edition (ICD-O-3) histology codes: 8000, 8010, 8140, 8144, 8210, 8211, 8213, 8220, 8221, 8255, 8260, 8261, 8262, 8263, 8480, and 8481.

### Data source

The NCDB is a joint project of the Commission on Cancer (CoC) of the American College of Surgeons (ACS) and the American Cancer Society [11]. The hospital-based cancer registry gathers data from over 1500 CoC-accredited facilities in the US capturing over 70% of colon cancer cases diagnosed annually [12].

### Variables and outcome measures

Patients and provider demographics were analyzed, including age, sex, race, insurance status, facility location, and facility site. Race data were further categorized in white, black, and American Indian/ Alaska Natives/ and Asian American/ Pacific Islander, hereafter AI/AN-API. For this work’s purpose, AI/AN and API races reported had low frequency and were merged into one category. The pathologic features analyzed included anatomic site (Colon, Rectum, Rectosigmoid Junction) and pathologic staging (per The American Joint Committee on Cancer, 7th Edition Staging Manual). The vital status and follow-up time from diagnosis to death/censor were also abstracted. The main outcome measure was the trend in CRC over time and potential cases without screening guideline amendment in the 45-to-50 population. Secondary outcome measures were the burden of CRC in the 45–50 population without the screening guideline amendment.

The CRC burden was calculated using CRC rates from 2004 through 2016 from the NCDB. The 2017 cases were excluded from the CRC burden calculation, as there was no vital status information published at the time of analysis. To estimate the age at death, the survival time was added to the patient’s age at diagnosis. The burden of CRC was quantified through the following standardized metrics: years lived with disability (YLD), years of life lost (YLL), disability-adjusted life years (DALY), and loss of future earnings. We utilized three recognized sequalae phases: (1) diagnosis/treatment, (2) metastatic/disseminated disease, and (3) palliative care/terminal disease, applying the respective disability weights to estimate the YLD for each case. The YLL was calculated based on the life expectancy value at the age of death at each case. The DALY is a sum of YLD and YLL. For this work, we utilized the Global Burden of Disease Cancer Collaboration published values for the three sequalae phases weights and the published values for the life expectancy according to the age at death [1, 13]. The estimation of the CRC economic burden was based on the estimated loss of future earnings due to cancer-related mortality [14]. Lost earnings were calculated multiplying the YLL by the adjusted published values of age-specific annual median earnings and age-specific full-time and part-time employment rates [14]. These values were adjusted for the consumer price index inflation rates, according to the respective year [15]. The average annual percentage change (AAPC) in the incidence of CRC from 2004 through 2017 was calculated for all cases and separately by sex, race, and anatomic site [4, 7]. Estimations for the CRC incidence from 2018 through 2030 were calculated assuming the AAPC for the 2004–2017 period will remain stable. The following calculation was used to estimate new cases over time:

$${\text{Cases}}\;\left( {{\text{n}}\;{\text{year}}} \right)\;{ = }\;{\text{Incidence}}\;{\text{of}}\;{\text{CRC}}\;\left( {{2}0{17}} \right)\; \times \;\left( {{\text{CRC}} \cdot {\text{AAPC}}} \right)/\left( {{1}00\; + \;{1}} \right)^{{{\text{n year}} - {2}0{17}}}$$

### Statistical analysis

The AAPC was calculated through jointpoint regression model, with up to two joint points (three segments) [16]. The best fit model was selected with the best fit model’s AAPC. In the zero jointpoint model, the AAPC equals one segment average percentage change (APC). In the one or two jointpoint model, the AAPC is the weighted average of each segment’s APC [17]. The trends were determined as increasing or decreasing when the AAPC was significantly different from zero, per two-sided statistical significance set at alpha < 0.05. No changes in the AAPC were considered stable trends (*p* ≥ 0.05). Analysis was performed with the Jointpoint Regression Program v4.9.0.0 (Statistical Methodology and Applications Branch, National Cancer Institute, Bethesda, MD) and figures with ggplot2 package from R Studio v1.4.1103 (Rstudio: Integrated Development Environment for R, Boston, MA).

## Results

From 2004 through 2017, there were 67,442 cases of colorectal adenocarcinoma in patients 45 to 50 years old. Of these, 54% (*n* = 36,417) were male and 46% (*n* = 31,025) female. The majority were white (78.3%, (*n* = 52,827). The most frequent site was the colon (59.7%, *n* = 40,247), followed by rectum (30%, *n* = 20,219) and then the rectosigmoid junction (10.3%, *n* = 6,976). From the surgical pathology, 26.5% (*n* = 17,862) had stage III and 21.1% (*n* = 14,200) had stage IV disease. Full demographic and pathologic data are in Table 1.

**Table 1:** Demographics and Pathologic Variables for CRC Cases in the 45-to-50 Age Group from 2004 through 2017

Variables	*N* = 67,442 (%)
Median Age (IQR) in years	47 (45–49)
Sex	
Female	31,025 (46)
Male	36,417 (54)
Race	
White	52,827 (78.3)
Black	10,129 (15)
Others	4,486 (6.7)
Insurance Type	
Private Insurance	48,984 (72.6)
Medicaid	7,482 (11.1)
Medicare	3,580 (5.3)
Uninsured	7,396 (11)
Facility Location	
Northeast	13,694 (20.3)
South	26,906 (39.9)
Midwest	16,372 (24.3)
West	10,470 (15.5)
Facility Type	
Community Cancer Program	6,548 (9.7)
Comprehensive Community Cancer Program	27,194 (40.3)
Academic Program	24,244 (35.9)
Integrated Network Cancer Program	9,456 (14)
Pathologic Stage	
0	5,951 (8.8)
1	9,630 (14.3)
2	11,581 (17.2)
3	17,862 (26.5)
4	14,200 (21.1)
Missing	8,218 (12.1)
Site	
Colon	40,247 (59.7)
Rectosigmoid Junction	6,976 (10.3)
Rectum	20,219 (30)

All values are counts (percentages) unless otherwise indicated, *CRC* colorectal cancer, *IQR* interquartile range

### Colorectal adenocarcinoma YLD, YLL, DALY, and lost earnings

From 2004 through 2016, there were 62,173 cases of CRC in the 45–50-year-old population. The overall mortality rate for this group was 34.1% (*n* = 21,216). Their CRC burden amounted to 899,905 DALY. The estimated YLD was 3% (*n* = 27,164) and the YLL was 97% (*n* = 872.741). Estimated overall lost earnings were $17 billion.

### AAPC and CRC future trends

**Fig. 1:**
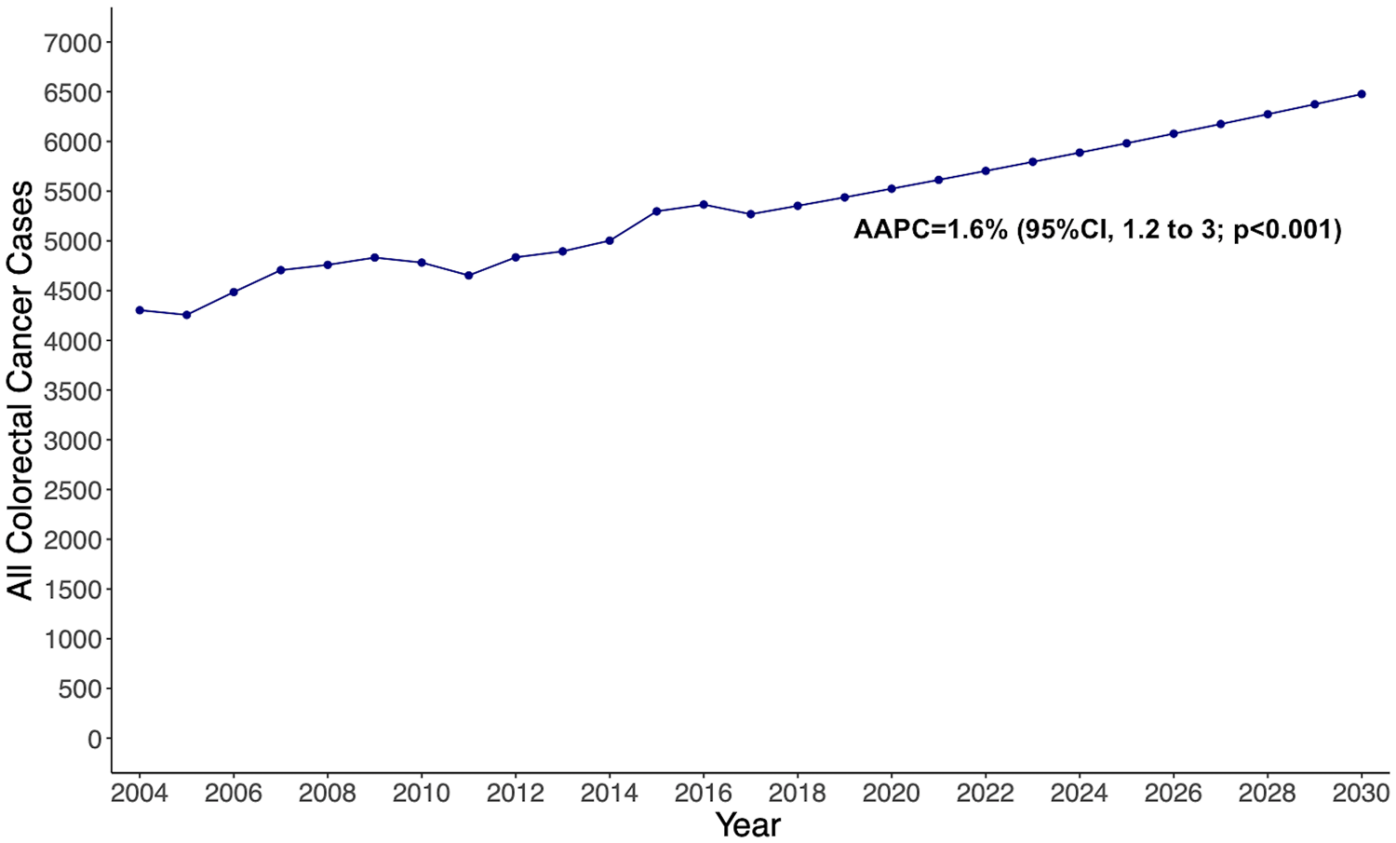
Estimated overall colorectal cancer cases from 2017 to 2030 among adults aged 45–50 years in the United States, based on Average Annual Percent Change (AAPC) from 2004 to 2017

**Fig. 2:**
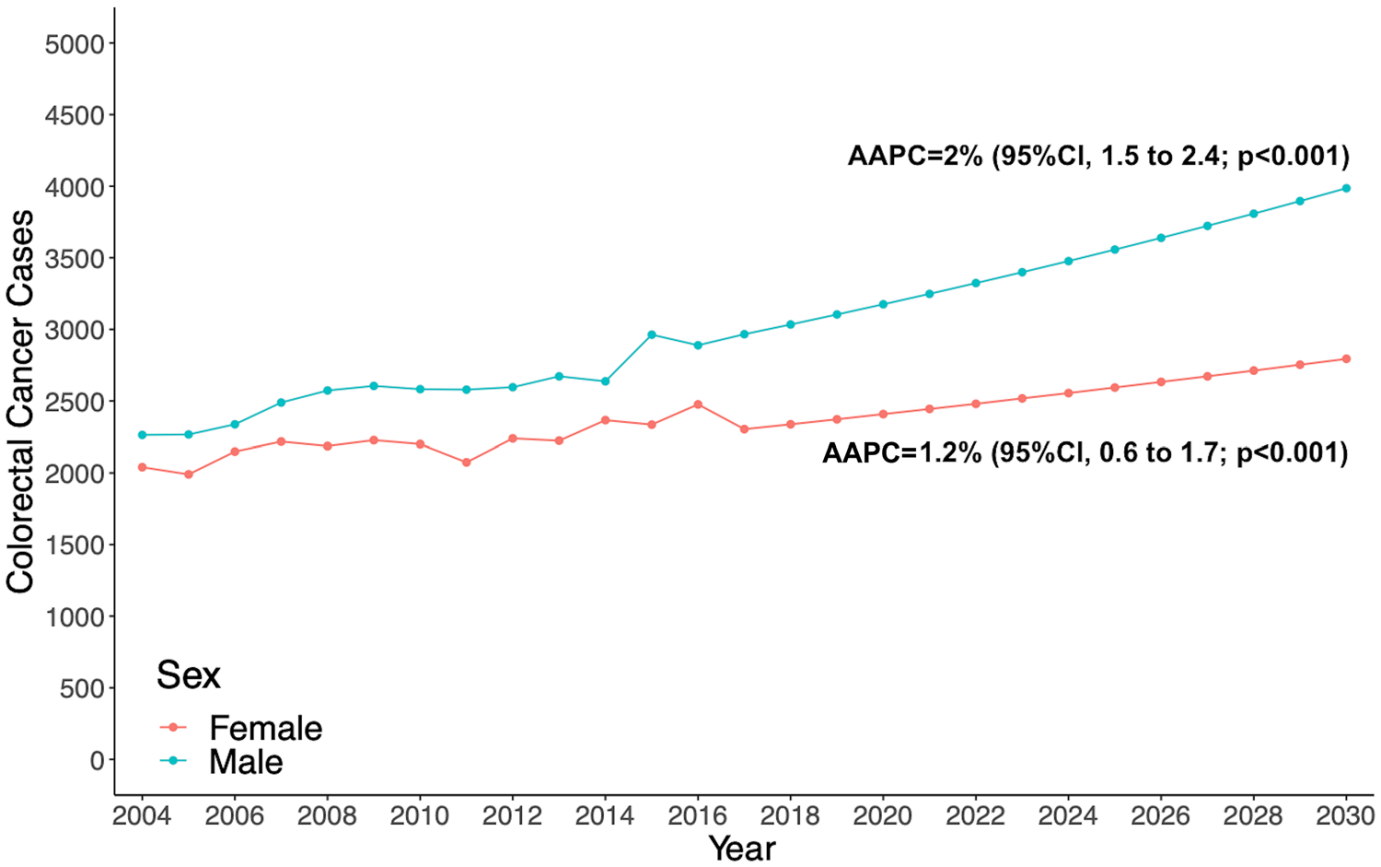
Estimated colorectal cancer cases, stratified by sex, from 2017 to 2030 among adults aged 45–50 years in the United States, based on Average Annual Percent Change (AAPC) from 2004 to 2017

From the jointpoint analysis, the overall incidence of CRC from 2004 through 2017 increased by an AAPC of 1.6% (95%CI, 1.2–2; *p* < 0.001), from 4303 cases in 2004 to 5259 cases in 2017 (Supplementary Fig. 1). Applying the 1.6% AAPC, it is estimated a 13-year increase of 25%, at 6,476 new cases by 2030. According to the estimations, from 2018 through 2030 there will be 76,676 new cases in the 45-to-50 years old (Fig. 1). In the sex-stratified analysis, the incidence of CRC in male patients increased by an AAPC of 2% (95%CI, 1.5–2.4; *p* < 0.001), from 2263 cases diagnosed in 2004 to 2,966 cases diagnosed in 2017 (Supplementary Fig. 2). According to our estimations, there will be a 30% increase in the 13-year period, at 3836 new cases by 2030. The incidence of CRC in female patients increased by an AAPC of 1.2% (95%CI, 0.6–1.7; *p* < 0.001) from 2040 cases diagnosed in 2004 to 2,303 cases in 2017. The 13-year period increase will be 17%, at 2689 cases by 2030 (Fig. 2).

**Fig. 3:**
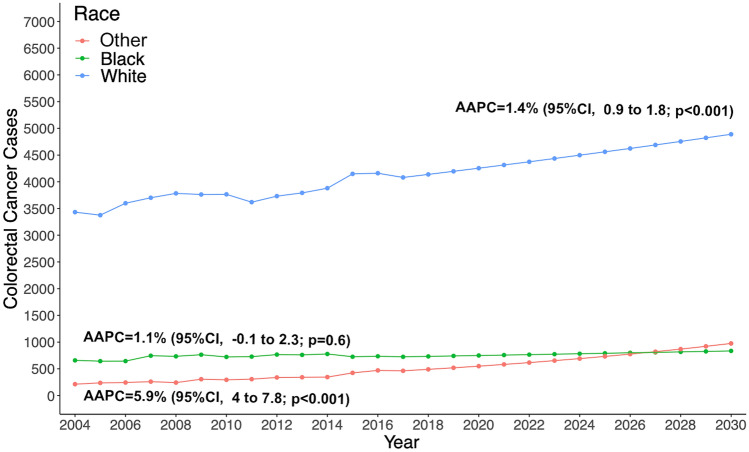
Estimated colorectal cancer cases, stratified by race, from 2017 to 2030 among adults aged 45–50 years in the United States, based on Average Annual Percent Change (AAPC) from 2004 to 2017

In the race-stratified analysis, the incidence of colorectal adenocarcinoma in white patients increased by an AAPC of 1.4% (95%CI 0.9–1.8; *p* < 0.001), from 3432 cases in 2004 to 4081 cases in 2017 (Supplementary Fig. 3). The estimated 13-year increase is 18%, at 4889 new cases by 2030. The incidence of colorectal adenocarcinoma was stable from 2004 through 2017 in black race patients (*p* = 0.6). The incidence of colorectal adenocarcinoma in other races increased by an AAPC of 5.9% (95%CI, 4–7.8; *p* < 0.001) from 2004 through 2017, from 213 cases in 2004 to 463 cases in 2017. There will be a 13-year increase of 110%, at 975 new cases diagnosed by 2030 (Fig. 3).

**Fig. 4:**
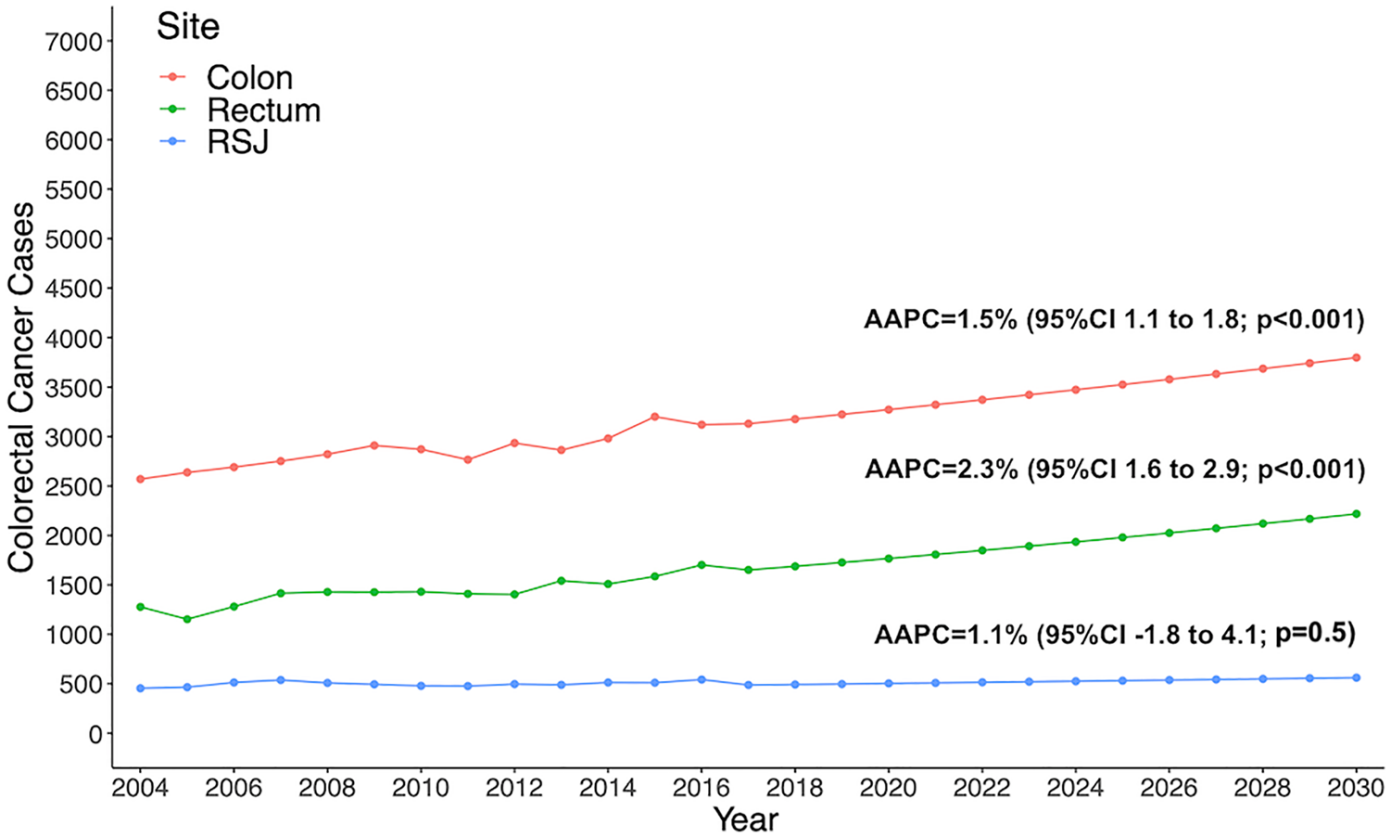
Estimated colorectal cancer cases, stratified by site, from 2017 to 2030 among adults aged 45–50 years in the United States, based on Average Annual Percent Change (AAPC) from 2004 to 2017. RSJ Rectosigmoid Junction

In dividing the analysis by site, the incidence of colon adenocarcinoma increased by an AAPC of 1.5% (95%CI, 1.1–1.8; *p* < 0.001), from 2570 cases in 2004 to 3120 cases in 2017 (Supplementary Fig. 4). The estimate 13-year increase is 21%, at an estimated 3798 new cases by 2030. The incidence of rectosigmoid junction adenocarcinoma was stable from 2004 through 2017 (*p* = 0.5). The incidence of rectal cancer increased by an AAPC of 2.3% (95%CI, 1.6–2.9; *p* < 0.001), from 1278 cases in 2004 to 1651 cases in 2017. The estimated 13-year increase is 31%, at 2218 new cases by 2030 (Fig. 4).

## Discussion

This work quantified the growth of CRC in the 45-to-50 population, finding an AAPC of 1.6% from 2004 through 2017. Without any changes, the incidence rates for CRC in the 45-to-50 population would increase 25% in a 13-year period, from 2017 to 2030. Additionally, sex, race, and anatomic site-specific variations in incidence were found within the 45-to-50 group. The greatest CRC increases were in American Indian/ Alaska Natives/ and Asian American/ Pacific Islander races, rectal cancer, and male sex.

This work uniquely quantifies the impact of CRC in the 45–50-year-old population, which has the potential change trends in the burden of CRC from the recent change in screening guidelines. The premature morbidity and mortality in this working age have major impact on the nation’s labor productivity, as this age contributes the greatest participation rates and productivity to the US work force [18]. Nearly half of the 45-to-50-year-old CRC cases presented with advanced stage disease, stage III or IV. These cases bear an inherently worse prognosis and subsequently a greater burden for this cohort [19–22]. Prior studies in other countries reported a substantial contribution from CRC premature mortality to earning losses. In Spain, the losses amounted to up to 10% or €510 million, of all cancer-related productivity losses in. In Ireland, these losses are estimated to accumulate to €8.3 billion between 2011 and 2030. Worldwide in 2017, CRC caused 19 million DALY, of which 95% were YLL [1, 23–25]. This is aligned with our findings of an impactful CRC burden of 899,905 DALY and massive lost earnings, resulting almost entirely from YLL. In an economic evaluation in the US, Bradley et al. assessed the cost savings from CRC screening in the overall population by 2020 could result in 101,353 deaths avoided and $33.9 billion in savings in reduced productivity loss, $12.4 billion from risk factor reduction, and $8.4 billion from improved treatment [26]. Thus, there is great potential benefit for the individuals and society in lowering the CRC screening age to 45 years old and targeting this population to ensure compliance.

The recently published USPSTF guidelines stated that there would be a moderate benefit in reducing CRC mortality and increasing life years gained in lowering the screening recommendation from 50 to 45 [8, 9]. Some evidence used by the USPSTF stems from a microsimulation model, where the benefits from lowering the initial screening age to 45 resulted in a moderate increase in life years gained and decrease in CRC cases and mortality, outweighing the estimated burden from screening [27]. They provided estimations for the general population at average risk, while the current work uses a retrospective review of actual hospital-based data. The findings in the current work agree and complement this modeling. We found missed opportunities for earlier diagnosis in almost half of the cohort. CRC is preventable and amenable to treatment if detected early and appropriate treatment delivered [28, 29]. Previous work has shown younger patients are more tolerant and more likely to receive appropriate treatment [30–32]. Thus, there is great potential benefits for the 45-to-50 cohort specifically from lowering the CRC screening recommendations.

The increase in CRC rates over time in individuals from 45-to-50 years old contrasts with the overall population trends. According to CRC statistics work in United States, the overall CRC rates have been declining since the mid-1980s, related to change in risk factors and CRC screening uptake. While the incidence rates for individuals 50 years old and younger had been increasing since the mid-1990s [10]. The current CRC 5-year AAPC for overall male population is − 1.5% and for female is − 1.0% [4].

The overall increase in EOCRC has been reported by other works.[2, 4, 33] This work is the 1^st^ to focus just on the 45–50-year-old population having an active intervention. Several published works in the overall EOCRC population agree with the findings here of higher incidence for males and certain racial minorities, but within all EOCRC ages.[10, 34] Published works also differ from the current work in demographic and anatomic variations. Siegel et al. reported EOCRC (20–49 years old) increased in young adults, with a ten-year AAPC from 0.8% to 4.2% (2.2% in United States) and no anatomic site-specific variation [2]. In New York State, Altieri et al. reported an increase in CRC for patients 21–50 years old, but with no association to race or gender [35]. Regardless of any heterogeneity in findings across studies, the common denominators driving the disparities in CRC trends are known risk factors and socio-economic factors. Alcohol consumption, smoking, obesity, sedentary lifestyle, and high-fat, processed diets, [36, 37] as well as access to convenient cost-effective, high-quality screening affect CRC trends [38]. Race is another factor impacting trends. In this work, there was an alarming increase in CRC rates in other patients in the 45-to-50 age group. Some racial disparities in CRC trends might be related to unmodifiable genetic factors. But race is also a social construct, grouped by differences in income, education, health insurance, and access to care and screening. The effectiveness of the screening recommendations could be lower in this population, and targeted interventions may enhance access and improve earlier-stage detection in the most vulnerable populations [10]. For instance, recognizing that African Americans were being diagnosed at a younger average with more advanced stage disease and had the highest CRC mortality, the American College of Gastroenterology (ACG) changed guidelines to recommend that African Americans should begin their CRC screening at age 45 [39]. While screening disparities still exist, CRC screening rates among African Americans have improved significantly since changing the initial guidelines. Programs that target individuals, their communities, address known barriers to screening, use multiple methods of message delivery, and are delivered over multiple time points in addition to earlier screening may have the best outcomes for reversing the trends seen within the 45-to-50 population [40, 41].

Although this work’s estimations comprehend a 13-year time frame, it is unlikely that the CRC rates in the 45-to-50-year-old population would decline or even reach a plateau, beyond this period, without any interventions. The largest population groups are the 20–39 years old, currently [42]. Considering the reported positive trends in CRC in this age bracket and this being the most populous group [4], we would expect to see rates continue to rise exponentially for the foreseeable future.

We recognize the limitations in the current work. The NCDB-PUF is a retrospective hospital-based registry, so there are limitations in the data fields available, risks for data entry errors, and assumptions made from the fields reported. However, the NCDB-PUF is a nationally representative, large sample with quality standards in data collection and reporting to maintain accreditation and systematically employed quality checks [12, 43]. These features yield a reliable and comprehensive hospital registry that can be used for cancer trend studies. Additional compensations as health insurance, retirement benefits, and paid leaves are not factored in the loss of future earnings calculation, what might result in underestimation. The possible benefits are also reliant on eligible patients having the appropriate screening. With the current rates of compliance with screening less than 2/3 in the general population, further work will need to assess effective screening uptake strategies.

In conclusion, CRC has been steadily increasing in the US in the 45-to-50 age group at an AAPC of 1.6%. Without any intervention, the incidence will continue to rise 25% through 2030. There is a huge economic and quality of life burden from CRC in this population. Thus, there is great potential benefit in lowering the recommended routine CRC screening age to 45. Higher rates of CRC incidence were seen within the 45-to-50 age group for male and AI/AN-API races. These disparities can guide targeted interventions to optimize the uptake of screening in these groups. In benchmarking the current and potential future CRC burden in this group, ongoing research can be designed to assess outcomes on the US incidence and survivorship from this change in screening guidelines. Additionally, this assessment could be useful in other countries as well. Having data are imperative for making evidence-based decisions for public health initiative. Even when national screening programs are in place, performing this exercise is important to ensure that the population is compliant and the methods selected for screening are effective.

## Supplementary Information

Below is the link to the electronic supplementary material.

Final selected model from Jointpoint regression analysis and Average Annual Percent Change (AAPC) for all colorectal cancer cases among adults aged 45 to 50 years from 2004 to 2017 from the National Cancer Database. Analysis was performed with the Jointpoint Regression Program v4.9.0.0 (Statistical Methodology and Applications Branch, National Cancer Institute, Bethesda, MD) (tiff 8340 KB)

Final selected model from Jointpoint regression analysis and Average Annual Percent Change (AAPC) for all colorectal cancer cases among adults aged 45 to 50 years from 2004 to 2017 from the National Cancer Database, stratified by sex. Analysis was performed with the Jointpoint Regression Program v4.9.0.0 (Statistical Methodology and Applications Branch, National Cancer Institute, Bethesda, MD) (tiff 3443 KB)

Final selected model from Jointpoint regression analysis and Average Annual Percent Change (AAPC) for all colorectal cancer cases among adults aged 45 to 50 years from 2004 to 2017 from the National Cancer Database, stratified by race. Analysis was performed with the Jointpoint Regression Program v4.9.0.0 (Statistical Methodology and Applications Branch, National Cancer Institute, Bethesda, MD) (tiff 6256 KB)

Final selected model from Jointpoint regression analysis and Average Annual Percent Change (AAPC) for all colorectal cancer cases among adults aged 45 to 50 years from 2004 to 2017 from the National Cancer Database, stratified by site. Analysis was performed with the Jointpoint Regression Program v4.9.0.0 (Statistical Methodology and Applications Branch, National Cancer Institute, Bethesda, MD) (tiff 6256 KB)
