# Supplementary figures and images for: Colorectal cancer in the 45-to-50 age group in the United States: a National Cancer Database (NCDB) analysis
Source: Surg Endosc. 2021 Dec 9;36(9):6629–37. doi: 10.1007/s00464-021-08929-6 (PMC9402772; doi:10.1007/s00464-021-08929-6)

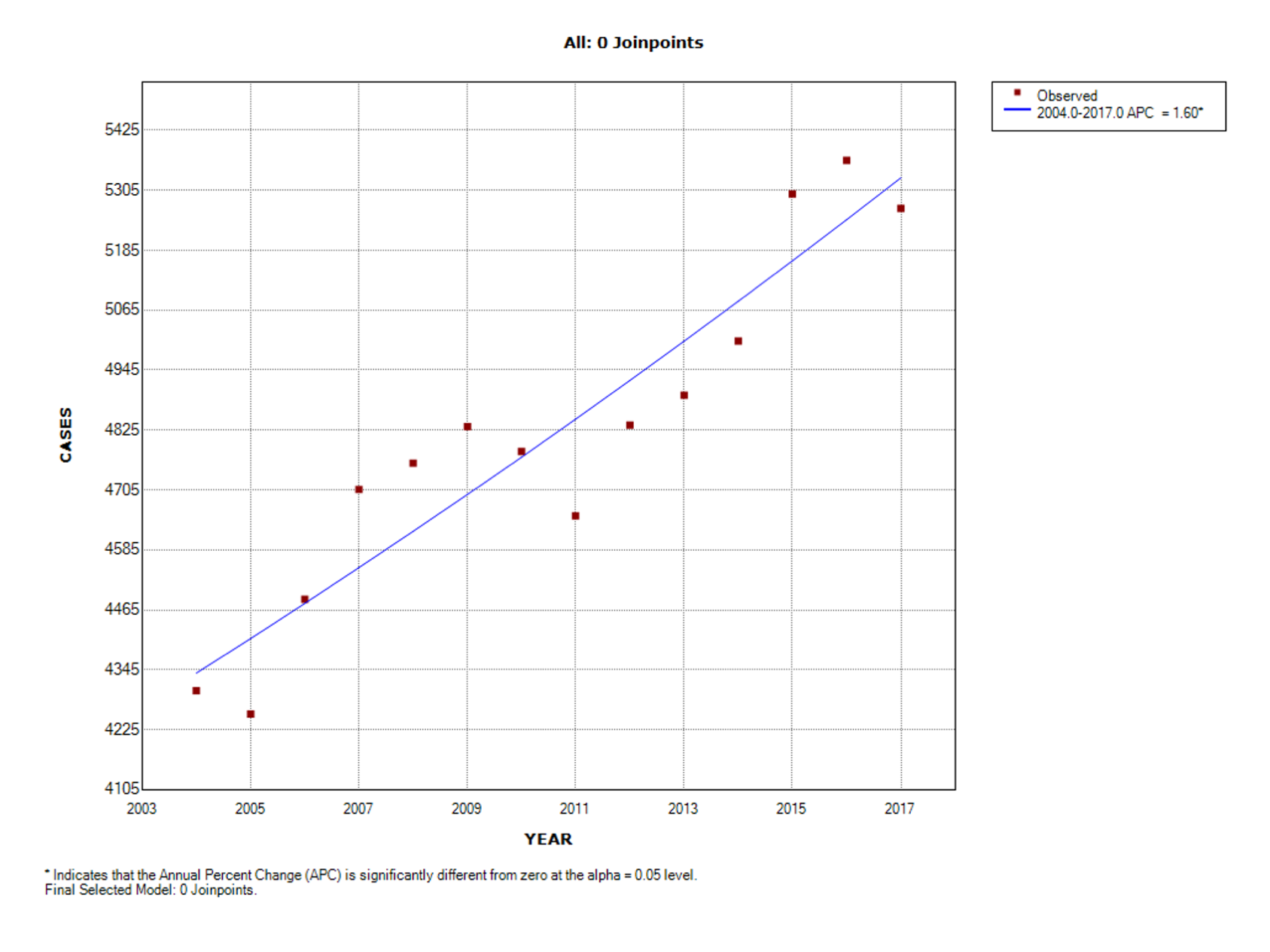

Supplement: Supplementary file 1 — Final selected model from Jointpoint regression analysis and Average Annual Percent Change (AAPC) for all colorectal cancer cases among adults aged 45 to 50 years from 2004 to 2017 from the National Cancer Database. Analysis was performed with the Jointpoint Regression Program v4.9.0.0 (Statistical Methodology and Applications Branch, National Cancer Institute, Bethesda, MD) (tiff 8340 KB) [file 464_2021_8929_MOESM1_ESM.tiff]

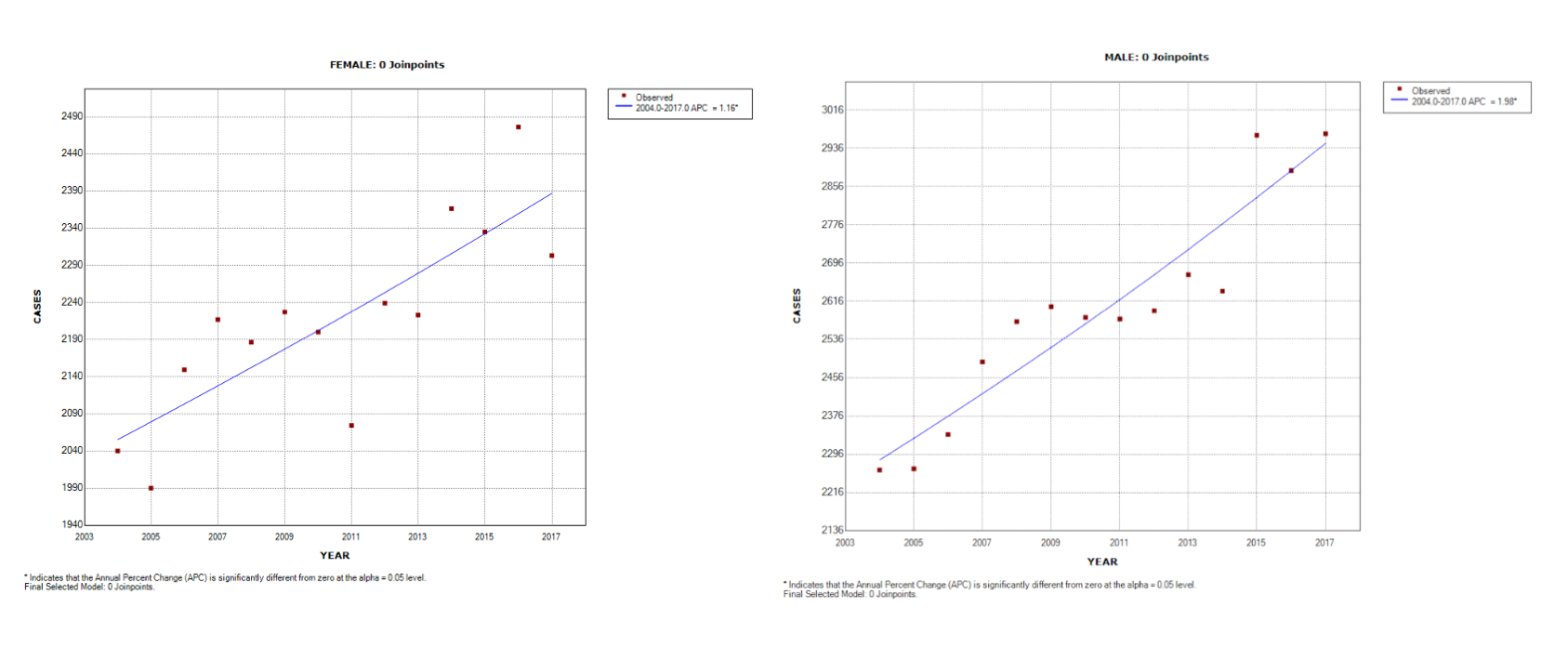

Supplement: Supplementary file 2 — Final selected model from Jointpoint regression analysis and Average Annual Percent Change (AAPC) for all colorectal cancer cases among adults aged 45 to 50 years from 2004 to 2017 from the National Cancer Database, stratified by sex. Analysis was performed with the Jointpoint Regression Program v4.9.0.0 (Statistical Methodology and Applications Branch, National Cancer Institute, Bethesda, MD) (tiff 3443 KB) [file 464_2021_8929_MOESM2_ESM.tiff]

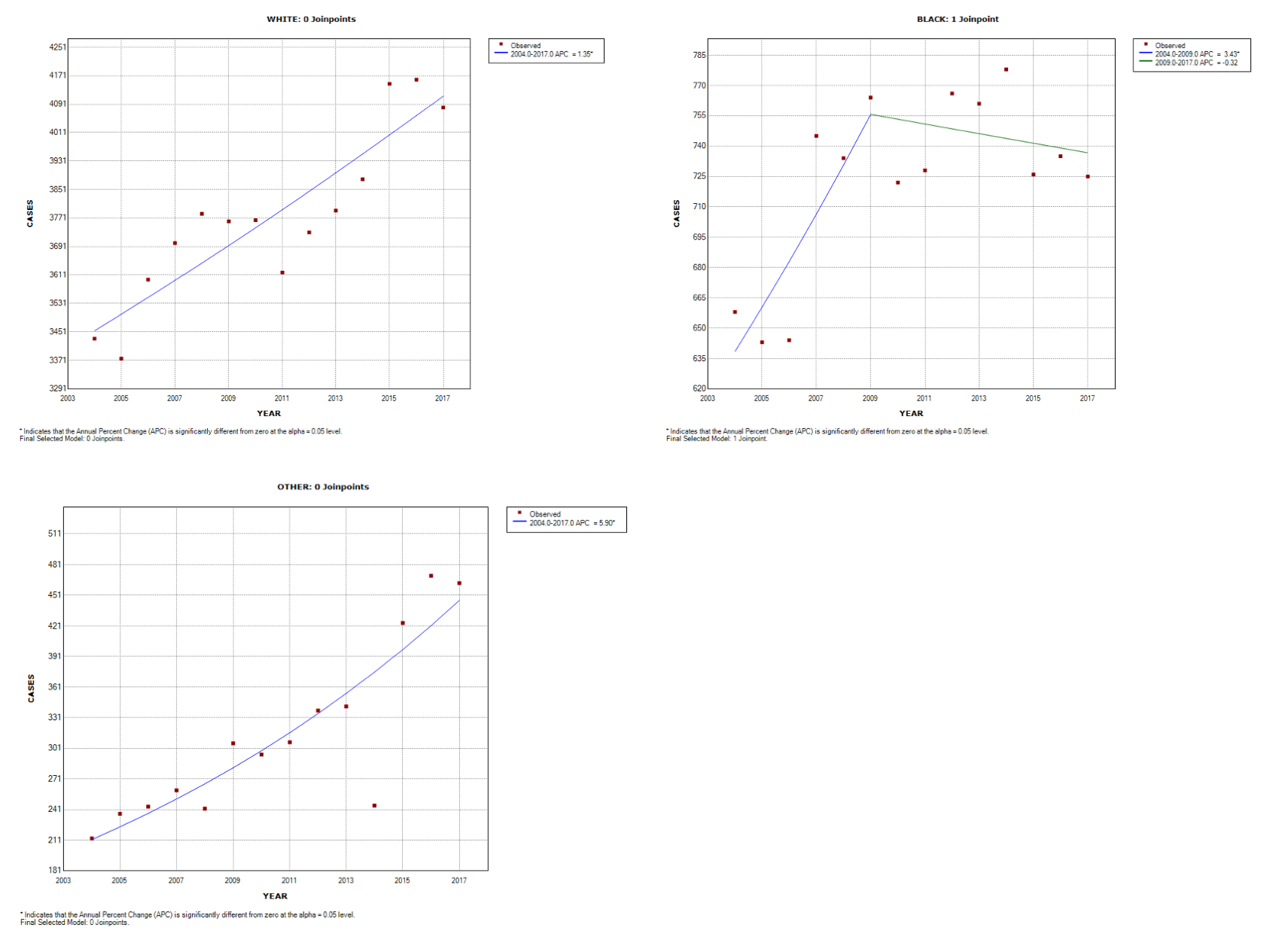

Supplement: Supplementary file 3 — Final selected model from Jointpoint regression analysis and Average Annual Percent Change (AAPC) for all colorectal cancer cases among adults aged 45 to 50 years from 2004 to 2017 from the National Cancer Database, stratified by race. Analysis was performed with the Jointpoint Regression Program v4.9.0.0 (Statistical Methodology and Applications Branch, National Cancer Institute, Bethesda, MD) (tiff 6256 KB) [file 464_2021_8929_MOESM3_ESM.tiff]

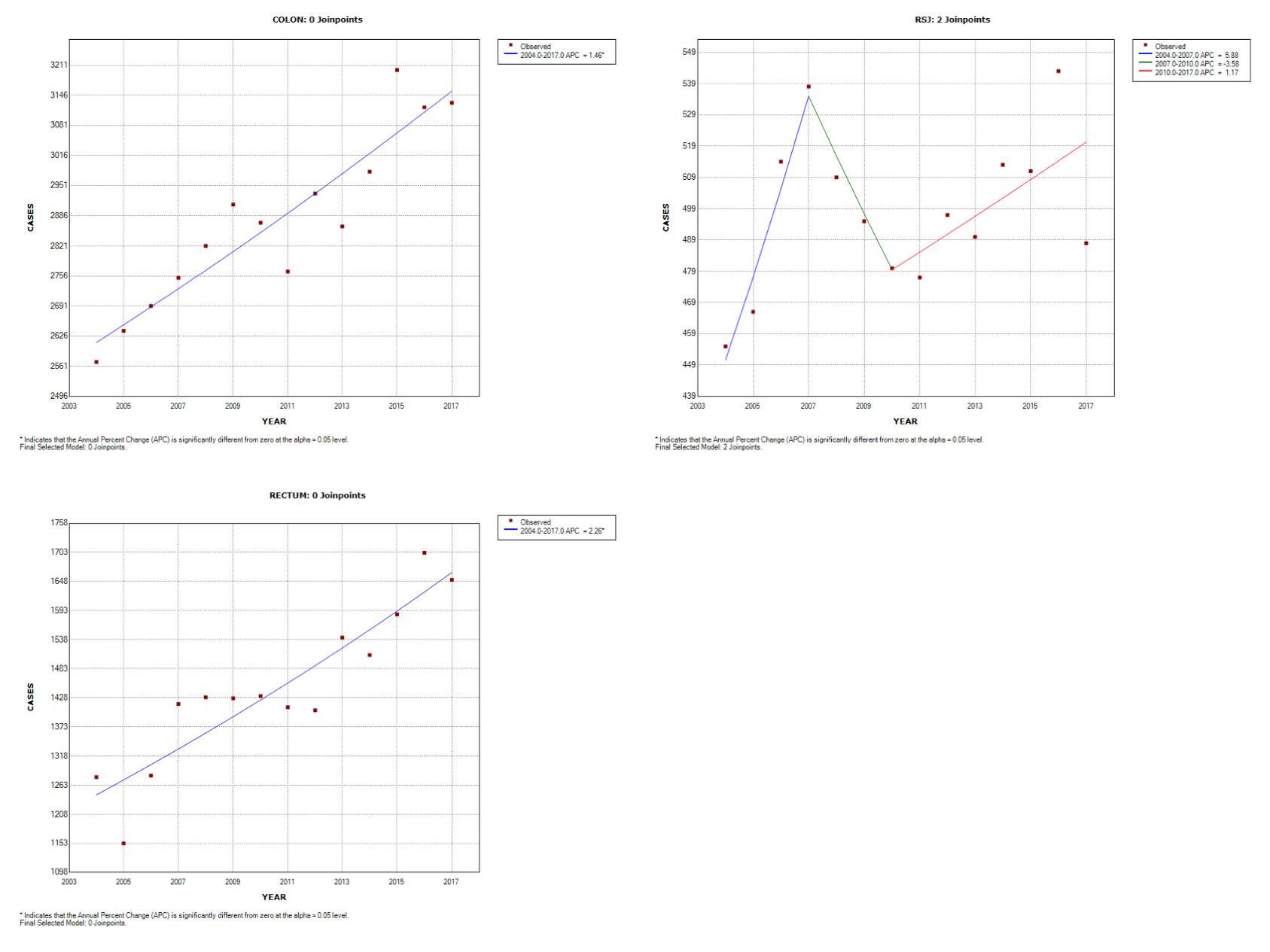

Supplement: Supplementary file 4 — Final selected model from Jointpoint regression analysis and Average Annual Percent Change (AAPC) for all colorectal cancer cases among adults aged 45 to 50 years from 2004 to 2017 from the National Cancer Database, stratified by site. Analysis was performed with the Jointpoint Regression Program v4.9.0.0 (Statistical Methodology and Applications Branch, National Cancer Institute, Bethesda, MD) (tiff 6256 KB) [file 464_2021_8929_MOESM4_ESM.tiff]
